# A Model-Based Joint Identification of Differentially Expressed Genes and Phenotype-Associated Genes

**DOI:** 10.1371/journal.pone.0149086

**Published:** 2016-03-10

**Authors:** Samuel Sunghwan Cho, Yongkang Kim, Joon Yoon, Minseok Seo, Su-kyung Shin, Eun-Young Kwon, Sung-Eun Kim, Yun-Jung Bae, Seungyeoun Lee, Mi-Kyung Sung, Myung-Sook Choi, Taesung Park

**Affiliations:** 1 Interdisciplinary Program in Bioinformatics, Seoul National University, Kwan-ak St. 599, Kwan-ak Gu, Seoul, Korea; 2 Department of Statistics, Seoul National University, Kwan-ak St. 599, Kwan-ak Gu, Seoul, Korea; 3 Center for Food and Nutritional Genomics Research, Department of Food Science and Nutrition, Kyungpook National University, Daegu, Korea; 4 Department of Food and Nutrition, Sookmyung Women’s University, Seoul, Korea; 5 Division of Food Science and Culinary Arts, Shinhan University, Gyeonggi, Korea; 6 Department of Mathematics and Statistics, Sejong University, Seoul, Korea; Queen's University Belfast, UNITED KINGDOM

## Abstract

Over the last decade, many analytical methods and tools have been developed for microarray data. The detection of differentially expressed genes (DEGs) among different treatment groups is often a primary purpose of microarray data analysis. In addition, association studies investigating the relationship between genes and a phenotype of interest such as survival time are also popular in microarray data analysis. Phenotype association analysis provides a list of phenotype-associated genes (PAGs). However, it is sometimes necessary to identify genes that are both DEGs and PAGs. We consider the joint identification of DEGs and PAGs in microarray data analyses. The first approach we used was a naïve approach that detects DEGs and PAGs separately and then identifies the genes in an intersection of the list of PAGs and DEGs. The second approach we considered was a hierarchical approach that detects DEGs first and then chooses PAGs from among the DEGs or vice versa. In this study, we propose a new model-based approach for the joint identification of DEGs and PAGs. Unlike the previous two-step approaches, the proposed method identifies genes simultaneously that are DEGs and PAGs. This method uses standard regression models but adopts different null hypothesis from ordinary regression models, which allows us to perform joint identification in one-step. The proposed model-based methods were evaluated using experimental data and simulation studies. The proposed methods were used to analyze a microarray experiment in which the main interest lies in detecting genes that are both DEGs and PAGs, where DEGs are identified between two diet groups and PAGs are associated with four phenotypes reflecting the expression of leptin, adiponectin, insulin-like growth factor 1, and insulin. Model-based approaches provided a larger number of genes, which are both DEGs and PAGs, than other methods. Simulation studies showed that they have more power than other methods. Through analysis of data from experimental microarrays and simulation studies, the proposed model-based approach was shown to provide a more powerful result than the naïve approach and the hierarchical approach. Since our approach is model-based, it is very flexible and can easily handle different types of covariates.

## Background

The development of new technologies has greatly affected the biological research field. Specifically, the advent of microarray technology provides a crucial turning point in biological research [1,2,3,4]. Microarray technology has commonly been used for simultaneously identifying the gene expression patterns in cells for thousands of genes. In addition, the sensitivity and specificity of microarray technology continues to improve, and microarrays are becoming a more economical research tool [5]. An important emerging medical application for microarray technology is clinical decision support for diagnosis of a disease as well as the prediction of clinical outcomes in response to a treatment [6].

Recently, the improvements in microarray technology have been guiding the development of various platforms. Many studies have tried to integrate several platforms; for example, the MicroArray Quality Control (MAQC) project provided gene expression levels that were measured from seven different platforms. The MAQC study provided a resource representing an important first step toward establishing a framework for the use of microarrays in clinical and regulatory settings [7]. In addition, microarray technology has been successfully commercialized, and as a result, a substantial amount of microarray data has been generated. Several studies have performed an integration analysis of microarray data. Meta-analysis is powerful for unifying the results of various gene expression studies (for example, breast cancer [8]). Statistical models such as analysis of variance are effective in integration analysis for identifying genes that have different gene expression profiles in the presence of many controlling variables [9].

In general, the primary goal of microarray data analysis is to identify differentially expressed genes (DEGs). Microarray technology allows us to obtain data on the expression of target genes more easily than other technologies. DEGs have become more easily detected by microarray technology than ever before. When applied to experimental data, the causal genes related to diseases can be obtained by discovering DEGs. Over the last decade, numerous statistical methods have been proposed such as t-tests, significance analysis of microarray (SAM) [10], regression modeling, mixed modeling [11], and local pooled error (LPE) tests [12].

Of these approaches, the t-test is the most popular statistical test for comparing the means between two groups. The t-test is a parametric method that requires a normality assumption. However, microarray data rarely satisfy the normal distribution assumption. Therefore, a permutation test that does not require such assumptions is preferably used to detect DEGs [13,14]. The SAM [10] uses a t-type of statistics using a fudge factor to stabilize the variance, and controls for the false discovery rate (FDR) [15]. The SAM is also a non-parametric analysis that does not require normality distributional assumption.

The application of microarray technology has also led to diverse studies that go beyond identifying DEGs such as a study examining the relationship between phenotype and expression data. Various phenotypes have been used in microarray experiments; for example, the survival time was utilized as a phenotype for analyzing the recurrence of cancer in clinical studies [16,17]. Several genes associated with the survival time were identified. Microsatellite instability (MSI) was utilized as a phenotype in a microarray study of colorectal cancer. Since the CpG island methylator phenotype (CIMP) was associated with MSI and BRAF mutations in colorectal cancer [18], MSI has played an important role in colorectal cancer studies. In addition, the tumor subtype can also be an important phenotype. For example, estrogen receptor(ER), progesterone receptor (PR), and *HER2* jointly define the subtypes of breast cancer. The triple-negative phenotype (ER-negative, PR-negative, and HER2-negative) is most commonly used [19].

Phenotype-associated genes (PAGs) are the genes that are associated with a phenotype of interest. The PAGs can be identified by regression analyses such as linear regression analysis for continuous phenotypes and the Cox regression model for survival time phenotypes [20]. When the phenotype is a binary variable representing two groups, the identification of PAGs becomes equivalent to identification of DEGs.

In this article, we focus on the joint identification of DEGs and PAGs in microarray data analyses. Our study was motivated by the need for an analysis of a microarray experiment consisting of high fat diet (HFD) and normal diet (ND) groups. Ten mice were assigned to each group for the microarray experiment. In addition, four phenotypes reflecting the expression levels of leptin, adiponectin, insulin-like growth factor 1 (IGF-1) and insulin were measured in blood samples. Leptin is an adipocyte-secreted hormone with a key role in energy homeostasis [21]. IGF-1 is similar in molecular structure to insulin and is an important hormone for childhood growth. Adiponectin controls glucose levels as well as fatty acid breakdown, and Insulin is one of the most important hormones in the metabolic system of mammals. The microarray experiment focused on gene expression changes associated with dietary fat control, and the determination of influential genes associated with obesity-related phenotypes. Thus, we need to identify DEGs for HFD and ND groups that are also PAGs for the four obesity-related phenotypes.

Although many approaches have been proposed for the separate identification of DEGs and PAGs, only a few approaches are available for the joint identification of DEGs and PAGs. The first approach we used for the joint identification of DEGs and PAGs was a naïve approach that detects DEGs and PAGs separately and then identifies the intersecting genes from the lists of PAGs and DEGs. The second approach is a hierarchical approach [22] that detects DEGs first and then chooses PAGs among DEGs or vice versa. Both approaches are two-stage analyses that require separate testing of DEGs and PAGs, making it difficult to control false positive errors.

We propose a new model-based approach for the simultaneous identification of DEGs and PAGs. Our model-based approach uses a linear regression model. We have use the linear regression model since it is easy to use, flexible in dealing with individual covariates, and easy extendibility (i.e. extension to permutation test can be done without using normality assumption). Our method is a one-stage analysis that takes less computing time, makes it easier to control false positive errors, and has greater power than naïve or hierarchical approaches. Through analysis of data from a microarray experiment carried out in mice and from simulation studies, we compare our model-based approach with naïve and hierarchical approaches.

## Method

### Ethics statement

All animal experimental procedures were reviewed and approved by the Institutional Animal Care and Use Committee of Sookmyung Women’s University (SMU-IACUC-2011-0401-005).

### Data

Microarray data consisted of data obtained from HFD and ND groups of mice to determine influential genes associated with obesity. Four-week-old male C57BL/6J mice were purchased from SLC Japan (Hamamatsu, Tokyo, Japan). Mice were housed in plastic cages (three to four mice per cage) under a constant temperature (23 ± 2°C) and humidity (50 ± 10%) with a 12-h light/dark cycle. Animals were allowed to acclimatize to the laboratory environment for 1 week before the experiment onset. The composition of experimental diet was based AIN-93G. The fat sources of normal diet (ND, 15% of fat calories) and high-fat diet (HFD, 45% of fat calories) were based on corn oil and lard. The reference we have used for such fat percentage definition can be seen in “A high-fat diet impairs neurogenesis: Involvement of lipid peroxidation and brain-derived neurotrophic factor” [23]. Fresh diet was provided every 2~3 days and mice had free access to water and food throughout all experiments. Animals were maintained for 8 weeks and were killed by CO_2_ inhalation at 13 week of age. At necropsy, blood and tissue samples were collected; serum samples were prepared by centrifugation of whole blood samples at 650 × g for 20 min and stored at -80°C until analysis; colon tissues were rapidly removed, immediately frozen in liquid nitrogen, and stored at -80°C until microarray analysis.

Illumina MouseRef-8 v1.1 Expression BeadChip was used in our microarray experiment. We observed changes in the gene expression pattern due to HFD-induced obesity. We assigned 10 mice to each ND group and HFD group. Then, three mice from the ND group and six mice from the HFD group were selected via QC for the microarray experiment, and each sample had 45281 probes.

Four phenotypes associated with regulating metabolism were extracted using levels of expression in the blood sample including leptin, adiponectin, insulin-like growth factor 1 (IGF-1), and insulin. Serum insulin concentration was measured with an ELISA kit (Linco Research, St Louis, MO, USA) according to the manufacturer’s instruction. Serum concentrations of IGF-1, leptin (R&D Minneapolis, MN, USA) and adiponectin (Biovendor, Brno, Czech Republic) were also measured with an ELISA kit, according to the manufacturer’s instructions. IGF-1 is similar in molecular structure to insulin and is an important hormone for childhood growth. Adiponectin controls glucose levels as well as fatty acid breakdown, and insulin is one of the most important hormones in the metabolic system of mammals. The expression values are log-transformed. After log-transformation, QQ plots and goodness of fit tests for normal distribution did not provide evidence that the data do not follow normal distribution. We provided Fig A in S1 File which shows p-values obtained by Shapiro Wilks tests performed to each gene expressions and also showed some QQ plots for genes which are significant from model-based approach in Fig B in S1 File.

### Detection of DEGs

First, we detected DEGs by using a two-sample t-test. Secondly, we used significance analysis of microarray (SAM) [10] for identifying DEGs. SAM uses the t- statistics modified by adding a fudge factor (s_0_) to common statistics as one of the penalty methods. The variable s_*i*_ is the estimated standard error from gene i, and s_0_ is calculated as a percentile based on α. Then, the following test statistic is used:

$${\text{d}}_{\text{i}}=\frac{\bar{Expression_{1i}}-\bar{Expression_{2i}}}{S_{i}+S_{0}},\;\text{i}=1,2,\ldots \;,\text{p}$$ (1)

In addition, the SAM method uses a permutation algorithm to control for the false discovery rate (FDR) [15]. Therefore, we can control FDR more easily with this test than for the other tests such as the t-test.

### PAGs detection

Linear regression analysis is utilized to determine PAGs. There are two treatment groups in our microarray data: ND and HFD. Group information is denoted by Group. Expression_i_ indicates the expression value for each gene. As mentioned earlier, the phenotypes of interest consist of leptin, adiponectin, IGF-1, and insulin expression. Linear regression analysis is performed for each phenotype. Two linear regression models are applied to identify the linear relationship between genes and phenotypes.

$$\text{M}1:\text{Phenotype}=\beta_{0}+\beta_{1}Expression_{i}+\epsilon_{i},\;\epsilon_{i}∼N\left(0,\sigma^{2}\right)$$ (2)

$$\text{M}2:\text{Phenotype}=\beta_{0}+\beta_{1}Expression_{i}+\beta_{2}Group+\epsilon_{i},\;\epsilon_{i}∼N\left(0,\sigma^{2}\right)$$ (3)

where i (= 1,2,…,p) represents the gene. Group information is denoted by Group. Expression_i_ indicates the expression value for each gene. The first model M1 is to identify the effect of expression on the phenotype, whereas the second model M2 is an extension of M1 with an additional Group covariate.

**Fig 1:**
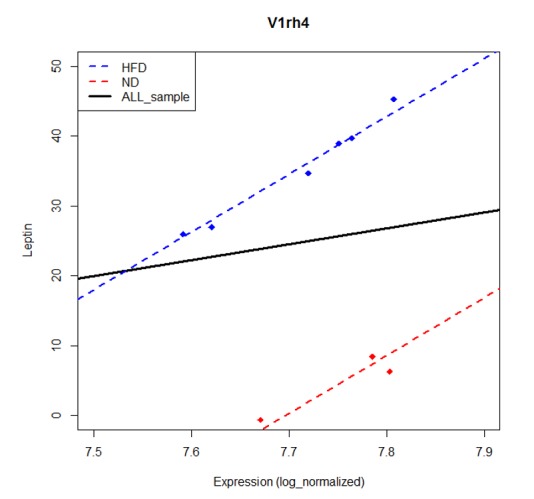
Model M1 and M2 problems. Model without considering a group effect cannot detect any significant correlation between Leptin and gene V1rh4. The y-axis represents the Leptin level and the x-axis the expression level of V1rh4. The blue line is a regression line for HFD, while the red line for ND. The black solid line is the regression line using all sample. However, if we consider the group effect, we can identify a significant association between phenotype and gene expression.

The significance of the linear relationship between gene and phenotype may be affected by the group effect because some genes may not have marginal effects on the phenotype but may have conditional effects given the group information. M1 is used for detecting the marginal effect, while M2 is used for detecting conditional effects. PAGs may depend on the group effect. For example, the v1rh4 gene is a non-PAG by model M1. However, it is identified as a PAG by model M2 (Fig 1). Model M2 is a more appropriate model than M1, when a group effect exists. However, model M1 provides PAGs that do not depend on the group effect, suggesting both M1 and M2 need to be fitted. Therefore, we use models M1 and M2 simultaneously to identify PAGs.

In model M1, the expression effect β_1_ is of the main interest. In model M2, β_1_ is still of the main interest even though the group effect β_2_ is added to explain the high fat diet effect between the ND group and the HFD group. The PAGs can be identified by testing the following hypotheses:

$${\text{H}}_{0}:\beta_{1}=0\;\text{for M}1$$ (4)

$${\text{H}}_{0}:\beta_{1}=0\;\text{for M}2$$ (5)

significances of these effects can be tested by calculating F-statistic for each gene.

### Joint identification analysis

#### Naïve approach

The naïve approach detects DEGs and PAGs separately, and then identifies the intersection of PAGs and DEGs. It consists of the following two steps:

1. Identifying DEGs set and PAGs set separately for total data.
2. Determining intersection gene sets of DEGs and PAGs.

#### Hierarchical approach

The hierarchical approach [21] detects DEGs first and then chooses PAGs among DEGs, or vice versa. The hierarchical approach consists of two steps: identifying DEGs and then detecting PAGs among the DEGs, or alternatively, identifying PAGs and then detecting DEGs among the PAGs.

1. Identifying DEGs by statistical tests such as two-sample *t*-tests, and SAM.
2. Detecting PAGs by linear regression models M1 and M2 for the DEGs selected at Step 1.

Both naïve and hierarchical approaches are two-stage analyses that require separate testing of DEGs and PAGs, and as a result, it was not straightforward to control the false positive errors because two tests are not independent. Thus, we propose a new model-based approach for joint identification of DEGs and PAGs. Our model-based approach uses a linear regression model, as explained in the following section.

#### Model-based approach

We propose a new test based on the likelihood ratio to determine DEGs and PAGs simultaneously. Consider the following model (6)

$$\text{M}3:\;Expression_{i}=\gamma_{0}+\gamma_{1}Group_{i}+\gamma_{2}Phenotype+\epsilon_{i}$$ (6)

For finding the DEGs and PAGs, consider the following hypotheses.

$${\text{H}}_{0}:\gamma_{1}=0\;or\;\gamma_{2}=0,{\text{H}}_{1}:\gamma_{1}\neq 0\;\&\;\gamma_{2}\neq 0$$ (7)

Under the null hypothesis, the likelihood can be maximized as follows.

$$argmax_{\gamma_{1},\gamma_{2\in {\text{H}}_{0}}}L_{0}(\gamma_{1},\gamma_{2})=argmax_{\gamma_{1},\gamma_{2}\in {\text{H}}_{0}}\text{max}(L(\gamma_{1},0),L(0,\gamma_{2}))$$ (8)

Thus, the likelihood ratio test statistic Λ is derived as follows:

$$\Lambda =-2\text{log}(\text{max}(L(\hat{\gamma_{1}}\prime ,0),L(0,\hat{\gamma_{2}}\prime ))/L(\hat{\gamma_{1}},\hat{\gamma_{2}}))$$ (9)

Where $\hat{\gamma_{1}}\prime$ is MLE of *γ*_1_ when *γ*_2_ = 0, $\hat{\gamma_{2}}\prime$ is MLE of *γ*_2_ when *γ*_1_ = 0 and ($\hat{\gamma_{1}},\hat{\gamma_{2}}$) are MLE in the whole parameter space

Un the null hypothesis, if *γ*_1_ or *γ*_2_ is large, the LRT statistic asymptotically follows χ^2^(1) distribution. Since this likelihood ratio test statistic is smaller than each likelihood ratio test statistic for testing H_01_: *γ*_1_ = 0 or H_02_: *γ*_2_ = 0, the p-value of LRT statistics is larger than that of both likelihood ratio test statistics for testing each beta. In this reason, even we test DEG and PAG at the same time with Chi-square test under 1 degree of freedom, type I error will be conserved. However, we cannot say that our test is most powerful among the tests. Thus, we performed simulation for power comparison between our proposed method and naïve approach.

#### Simulation study

For a more systematic comparison, we performed an extensive simulation study with following settings. First, we generated phenotype data from the normal distribution with the same mean and variance as the actual phenotype data. Since adiponectin most closely followed the normal distribution according to the normality test [24], the mean 25.06 and the variance 274.896 of adiponectin were used to simulate the phenotype data from the normal distribution. 50 samples consisting of 25 cases and 25 controls were generated. Finally, expression levels were generated from the Eq (6), where ε follows a normal distribution with mean 0 and variance 78.3.

## Results

### DEGs

Two-sample t-tests, and the SAM method, were utilized to identify DEGs. The results of t-tests were not significant after controlling for multiple testing at 5% level using Bonferroni correction or FDR. The SAM method provided significant results at the 0% median FDR = 0: *Tfrc*, *Sprr1a*, *Cyp4f16*, and *9030605I04Rik*. Although SAM provided only a few genes that are all up regulated genes, the SAM result is expected to be very reliable because it did not contain any false positives (FDR = 0)[24]. In Table 1, we displayed top 5 genes by using the nominal p-values from the Student’s t-test without any corrections for multiple comparisons. These lists of genes from two sample t-tests are different from those of SAM.

**Table 1:** Top gene list from t-test.

Gene Symbol	p-value	q-value
A230069A22Rik	0.000166	0.930077
2610018G03Rik	0.000190	0.930077
Pim3	0.000303	0.930077
D930042N17Rik	0.000326	0.930077
Ctns	0.000333	0.930077

### PAGs

**Fig 2:**
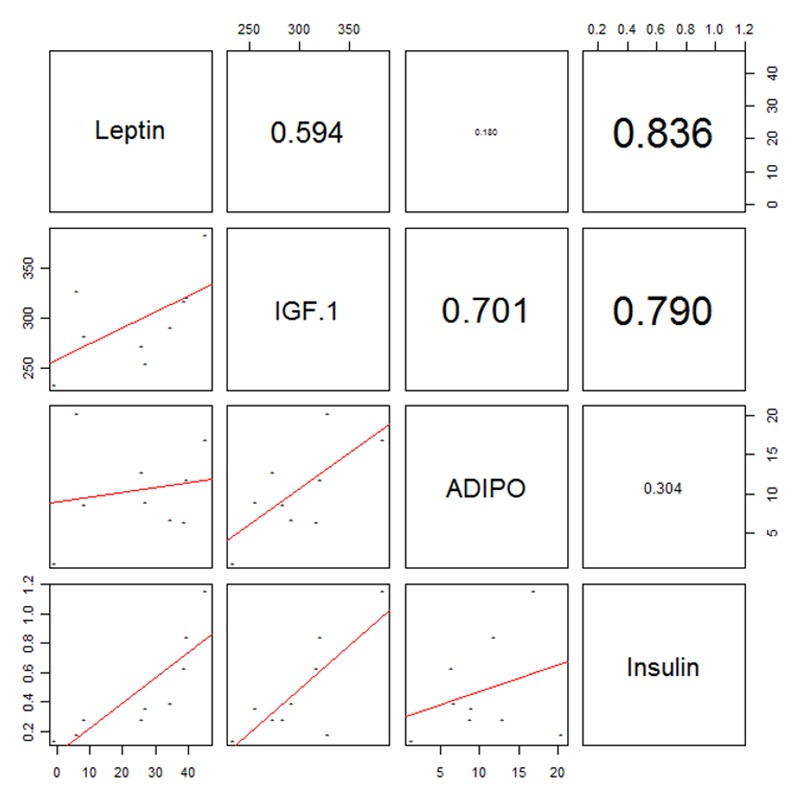
Correlation plot of phenotype. Leptin and insulin show the highest correlation value. IGF-1 and ADIPO give low correlation values with leptin and insulin, respectively.

Fig 2 shows a pairwise plot that illustrates the correlation coefficients among four phenotypes. All of them are highly positively correlated. Specifically, leptin and insulin have a particularly high correlation coefficient (0.836). Both leptin and insulin are known to be associated with body composition [25], BMI, and type 2 diabetes [26,27].

**Fig 3:**
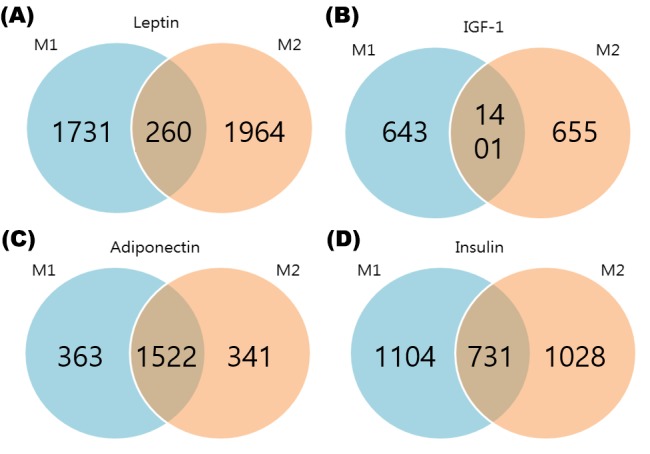
The Venn diagram of PAG. Fig (A) shows detected genes that are significant with leptin. Fig (B) shows detected genes that are significant with IGF-1. Fig (C) shows detected genes which are significant with ADIPO. Fig (D) shows detected genes that are significant with insulin. In all of these four figures, Both M1 and M2 reveal a large number of different significant PAGs. Thus, we could assume that conditional distributions of expression levels, given group, are different from marginal distribution in many genes.

**Fig 4:**
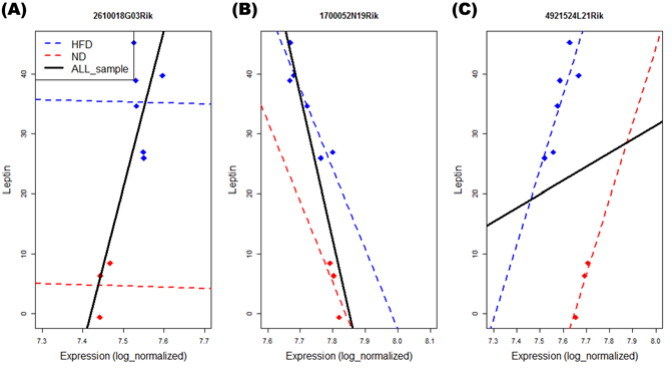
The PAG example plots: The y-axis is Leptin levels and the x-axis is the expression values of selected genes. Fig (A) shows an example of a gene that is significantly detected only by model M1 (1) (2610018G03Rik). Fig (B) shows an example of significantly detected gene by both models M1 and M2 (2) (1700052N19Rik). Fig (C) illustrates a gene significantly detected by model M2 (4921524L21Rik) only. Blue line is high fat diet (HFD) group and red line is normal diet (ND) group.

Models M1 and M2 were employed to detect PAGs for these phenotypes. Fig 3 shows the Venn diagram for the number of PAGs identified by M1 and M2 at the 5% significance level. Depending on the phenotypes, the numbers of overlapping and non-overlapping PAGs differ greatly. Thus, we could assume that in many genes, conditional distributions of gene expressions given groups are different from marginal distribution. Fig 4 shows examples of PAGs. Fig 4(A) shows example of pattern which was detected only by M1, Fig 4(B) shows that detected by both M1 and M2, and the Fig 4(C) shows that was detected only by M2.

### Joint identification analysis

We applied three joint identification methods to the microarray data from the mice. We first applied the naïve approach by comparing the list of DEGs and the list of PAGs. We then applied the hierarchical approach by detecting DEGs first and then PAGs. Finally, we applied the model based approach using the likelihood ratio test from model M3. For the purpose of a fair comparison, we fixed significance and FDR at the same level. Table 2 summarizes the numbers of significant genes that are both DEGs and PAGs. A larger number of significant genes were identified by the model based approach than the other methods. The top gene list for four phenotypes at a significance level of 5%, the p-value and q-value for each effect are summarized in Table 2. Since naïve approach and hierarchical approach provided the same results, the numbers of significant genes in Table 2 are the same for both approaches.

**Table 2:** Result of joint identification.

		Naïve approach	Naïve approach	Hierarchical approach (DEGs→PAGs)	Hierarchical approach (DEGs→PAGs)	Model-based approach
	DEGs method	M1	M2	M1	M2	M3
**Leptin**	**p-value (FDR)**	935(0)	124(0)	935(0)	124(0)	3787(15)
**IGF.1**	**p-value (FDR)**	41(0)	67(0)	41(0)	67(0)	1418(0)
**Adiponectin**	**p-value (FDR)**	0(0)	13(0)	0(0)	1(0)	1027(3)
**Insulin**	**p-value (FDR)**	337(0)	129(0)	337(0)	129(0)	1953(3)

The number of significant genes determined from three joint identification methods. The numbers in each cell show the number of significant genes without multiple comparison corrections. For the purpose of comparing each approach, significance level is fixed at 5%.The numbers in the brackets show the number of significant genes detected by FDR q-value below 5%. The model-based approach provides better power than other approaches.

### Simulation study

To check the type I error and power, we set the values of γ_1_ as follows: 0, 0.08, 0.12, 0.16, 0.2, 0.24, 0.28, and let γ_2_ vary from 0 to 8 by 2. These parameter values represent the effect sizes derived from the real data. Since our type I error needs to be evaluated not at a point but two axes generated by γ_1_ and γ_2_, we checked both (γ_1_ = 0, γ_2_≠0) and (γ_1_≠0, γ_2_ = 0) cases. Table 3 shows how well each method preserves the type I error under the various simulation settings. When both γ_1_ and γ_2_ equal 0, the type I error is smaller than 0.05 for all methods. However, as either γ_1_ or γ_2_ increases, the type I error becomes closer to 0.05. Therefore, all methods tend to preserve the type I errors.

**Table 3:** Type I error of the simulation results from naïve approach, hierachical approach and model-based approach.

		Naïve approach	Naïve approach	Hierarchical approach (DEGs→PAGs)	Hierarchical approach (DEGs→PAGs)	Model-based approach
γ1	γ2	M1	M2	M1	M2	M3
**0**	**0**	0.002	0.002	0.002	0.002	0.003
**0**	**2**	0.011	0.011	0.011	0.011	0.014
**0**	**4**	0.034	0.034	0.034	0.034	0.038
**0**	**6**	0.041	0.041	0.041	0.041	0.048
**0**	**8**	0.052	0.052	0.052	0.052	0.056
**0.08**	**0**	0.018	0.018	0.018	0.018	0.021
**0.12**	**0**	0.032	0.032	0.032	0.032	0.036
**0.16**	**0**	0.038	0.038	0.038	0.038	0.034
**0.2**	**0**	0.042	0.042	0.042	0.042	0.038
**0.24**	**0**	0.059	0.059	0.059	0.059	0.053
**0.28**	**0**	0.047	0.047	0.047	0.047	0.058

**Fig 5:**
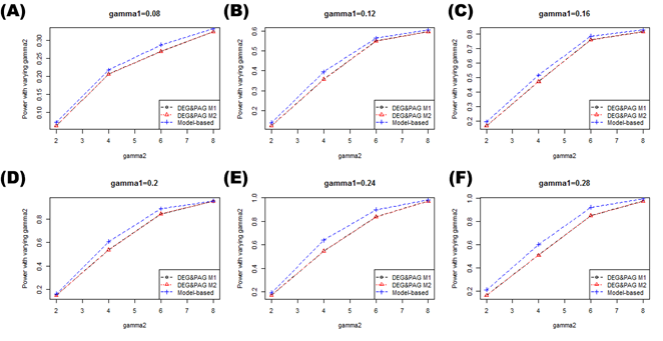
Power comparison and false positives plots with fixed γ_1_. These plots show the power comparisons between the methods while changing the γ_2_ with fixed γ_1._ Figs (A-F) shows the powers of each methods when γ_1_ changes from 0.08 to 0.28 by 0.04.

**Fig 6:**
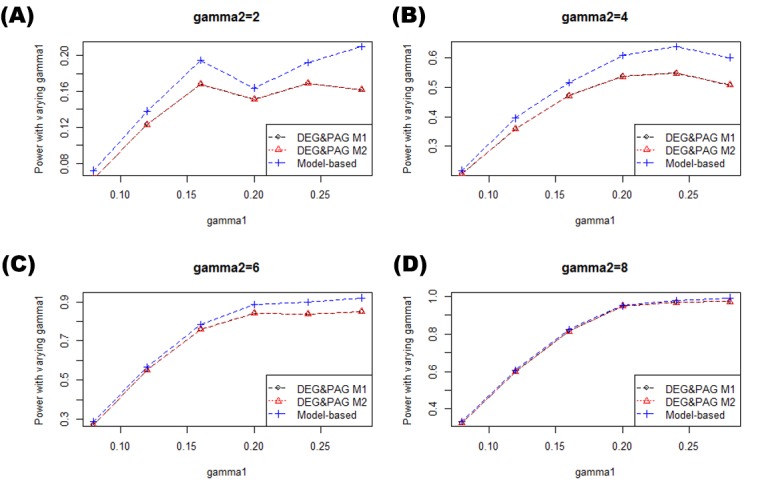
Power comparison and false positives plots with fixed γ_2_. These plots show the power comparisons between the methods while changing the γ_1_ with fixed γ_2._ Figs (A-F) shows the powers of each methods when γ_2_ changes from 2 to 8 by two.

Figs 5 and 6 show the powers of each methods for varying simulation settings. In this paper, power means the probability that the statistical hypothesis test is rejected when the null hypothesis is truly positive In Fig 5 where we fixed γ_1_ and varied γ_2_, M1 and M2 showed almost the same power in every case. On the contrary, the model-based method showed better power than other methods. In Fig 6 where we fixed γ2 and varied γ1, similar tendencies were observed.

## Discussion

As the experimental designs using microarrays becomes more complex, a more complicated analysis method needs to be developed. In early microarray studies, either DEGs or PAGs need to be identified. However, recent microarray designs require a more complicated method to detect the genes that are simultaneously DEGs and PAGs.

Our proposed model provided some interesting lists of genes (Table 4). Gene expression profiling figures are included in S1 File (Figs C-E). We also provided gene expressions for lists of genes and phenotypes for real data analysis in S2 and S3 Files. The four selected phenotypes are known to be associated with regulating metabolism. Therefore, many of the significant genes tend to be associated with this regulatory function. For example, The Grb10 gene is known to encode a growth factor receptor-binding protein that interacts with insulin receptors and insulin-like growth-factor receptors [28]. *FABP9 (Fatty Acid Binding Protein 9*, Testis) is a Protein Coding gene which is associated with sporadic breast cancer. GO annotations related to this gene include transporter activity and lipid binding [29]. *SGCE (Sarcoglycan*, *Epsilon*) is a Protein Coding gene which is associated with myoclonic, dystonia-11, or dystonia. It is known that *SGCE* gene works together with *SGCA* [30]. *SGCA* is known that is associated with type 2d [31]. Although *SGCA* cannot reach q-value below 0.05, that of p-value for model-based approach is 0.0011. Therefore, these genes would have significant relationships for finding differences of effects between high-fat diet and low-fat diet. *LGALS4 (Lectin*, *Galactoside-Binding*, *Soluble*, *4)* is a Protein Coding gene. Diseases associated with LGALS4 include colon adenocarcinoma and measles. Among its related pathways are Cell adhesion_Cell-matrix glycoconjugates. GO annotations related to this gene include carbohydrate binding [32].

**Table 4:** P-values and q-values of the top genes list from model based approach with 4 phenotypes.

Gene symbol	DEG	PAG M1 (p-value)	PAG M2(p-value)	model-based approach (p-value)	model-based approach(q-value)	phenotype
**Fabp9**	0.103	0.806	0.001	1.15E-05	0.037	leptin
**Aps-pending**	0.078	0.566	0.000	6.44E-07	0.015	leptin
**Sgce**	0.919	0.365	0.000	2.81E-06	0.024	leptin
**IGHV1S134_AF304554**	0.154	0.893	0.000	1.36E-06	0.021	leptin
**LOC434960**	0.326	0.879	0.001	1.07E-05	0.037	leptin
**Hspa12a**	0.346	0.081	0.000	7.51E-06	0.037	leptin
**Acd**	0.222	0.831	0.000	3.84E-06	0.025	leptin
**Lgals4**	0.524	0.124	0.000	9.69E-06	0.037	leptin
**Dll4**	0.177	0.721	0.000	8.86E-06	0.037	leptin
**V1rh4**	0.454	0.779	0.000	2.12E-07	0.010	leptin
**1700028I16Rik**	0.210	0.027	0.000	3.21E-06	0.024	leptin
**6330565C02Rik**	0.456	0.085	0.000	1.46E-05	0.044	leptin
**LOC231501**	0.365	0.043	0.000	6.36E-06	0.036	leptin
**Telo2**	0.461	0.770	0.000	9.01E-06	0.037	leptin
**Gm1549**	0.700	0.532	0.000	2.90E-06	0.024	leptin
**1700072E05Rik**	0.063	0.152	0.000	1.56E-06	0.024	adiponectin
**Asb17**	0.115	0.057	0.000	7.49E-07	0.018	adiponectin
**LOC236170**	0.308	0.010	0.000	7.87E-07	0.018	adiponectin
**9030421J09Rik**	0.040	0.513	0.000	1.19E-06	0.031	Insulin
**4930404F20Rik**	0.312	0.120	0.000	2.95E-06	0.045	Insulin
**Klhdc4**	0.475	0.065	0.000	1.38E-06	0.031	Insulin

These candidate genes demonstrate that the proposed method successfully identified the genes known to be associated with regulating metabolism. In addition, we have performed gene ontology analysis using DAVID functional annotation tool, using the genes only detected by model-based method with q-value below 0.05 as input. We found five GO terms contained in the CC_FAT terms that reached p-values below 0.05 or around 0.05 [33,34]. These results are summarized in Table 5. On the contrary, other methods could not find any significant molecular signatures. This may infer that genes undetected by the naïve and the hierarchical approaches could play important roles; in other words, important candidate genes may be overlooked if only the traditional approaches are used.

**Table 5:** Results of Gene ontology analysis for CC_FAT GOTERM using the functional annotation tool: Database for Annotation, Visualization and Integrated Discovery (DAVID).

Term	Count	%	p-value
**GO:0000781~chromosome, telomeric region**	2	11.76471	0.010036
**GO:0005887~integral to plasma membrane**	3	17.64706	0.032798
**GO:0031226~intrinsic to plasma membrane**	3	17.64706	0.035245
**GO:0043232~intracellular non-membrane-bounded organelle**	4	23.52941	0.078055
**GO:0043228~non-membrane-bounded organelle**	4	23.52941	0.078055

In this paper, we propose a statistical model for simultaneously detecting DEGs and PAGs. The proposed model is more efficient than other naïve methods for the joint identification of DEGs and PAGs. Using microarray data from an experiment and using simulation studies, the proposed model was compared to the other tested methods and was shown to have greater power. In other words, the proposed model identified more significant genes than other approaches under the same conditions (Table 4). The proposed approach was flexible and easy to extend. Since our model is a linear regression model, it can be extended for the cases where there are multiple factors. For example, our model can be applied to the analysis of a variety of covariates at the same time.

## Supporting Information

S1 File For checking the normality of log-transformed gene expressions, we performed Shapiro-Wilks test for each gene and drew a histogram of the p-values. P-values for most genes are larger than 0.05 which means log-transformed gene expressions follow normal distributions (**Fig A**). QQ-plots for some genes which are significant in model-based approach (**Fig B**). Gene expression profiles for significant genes with leptin detected by model-based approach. Blue dots are ND samples and Red dots are HFD samples. X-axis shows log-normalized gene expression for each gene and Y-axis shows expression of leptin (**Fig C**). Gene expression profiles for significant genes with adiponectin detected by model-based approach. Blue dots show ND samples and Red dots show HFD samples. X-axis shows log-normalized gene expression for each gene and Y-axis shows expression of adiponectin (**Fig D**). Gene expression profiles for significant genes with insulin detected by model-based approach. Blue dots are ND samples and Red dots are HFD samples. X-axis shows log-normalized gene expression for each gene and Y-axis shows expression of insulin (**Fig E**).
(PPTX)

S2 File Expression data for significant genes listed on Table 4. (CSV)

S3 File Phenotype data for real data analysis. (CSV)

## References

[1] van Hal NL, Vorst O, van Houwelingen AM, Kok EJ, Peijnenburg A, Aharoni A et al The application of DNA microarrays in gene expression analysis. Journal of Biotechnology. 2000 3 31;78(3):271–80. 10751688 10.1016/s0168-1656(00)00204-2

[2] Schulze A, Downward J. Navigating gene expression using microarrays—a technology review. Nature cell biology. 2001 8 1;3(8):E190–5. 11483980 10.1038/35087138

[3] Debouck C, Goodfellow PN. DNA microarrays in drug discovery and development. Nature genetics. 1999 1 1;21:48–50. 9915501 10.1038/4475

[4] Gershon D. Microarray technology: an array of opportunities. Nature. 2002 4 25;416(6883):885–91. 11976691 10.1038/416885a

[5] Heller MJ. DNA microarray technology: devices, systems, and applications. Annual review of biomedical engineering. 2002 8;4(1):129–53. 10.1146/annurev.bioeng.4.020702.153438 12117754

[6] Statnikov A, Aliferis CF, Tsamardinos I, Hardin D, Levy S. A comprehensive evaluation of multicategory classification methods for microarray gene expression cancer diagnosis. Bioinformatics. 2005 3 1;21(5):631–43. 15374862 10.1093/bioinformatics/bti033

[7] Shi L, Reid LH, Jones WD, Shippy R, Warrington JA, Baker SC, et al The MicroArray Quality Control (MAQC) project shows inter-and intraplatform reproducibility of gene expression measurements. Nature biotechnology. 2006 9 1;24(9):1151–61. 16964229 10.1038/nbt1239 PMC3272078

[8] Wirapati P, Sotiriou C, Kunkel S, Farmer P, Pradervand S, Haibe-Kains Bet al. Meta-analysis of gene expression profiles in breast cancer: toward a unified understanding of breast cancer subtyping and prognosis signatures. Breast Cancer Res. 2008 7 28;10(4):R65 10.1186/bcr2124 18662380 PMC2575538

[9] Park T, Yi SG, Shin YK, Lee S. Combining multiple microarrays in the presence of controlling variables. Bioinformatics. 2006 7 15;22(14):1682–9. 16705015 10.1093/bioinformatics/btl183

[10] Tusher VG, Tibshirani R, Chu G. Significance analysis of microarrays applied to the ionizing radiation response. Proceedings of the National Academy of Sciences. 2001 4 24;98(9):5116–21. 10.1073/pnas.091062498 PMC33173 11309499

[11] Pan W. A comparative review of statistical methods for discovering differentially expressed genes in replicated microarray experiments. Bioinformatics. 2002 4 1;18(4):546–54. 12016052 10.1093/bioinformatics/18.4.546

[12] Jain N, Thatte J, Braciale T, Ley K, O'Connell M, Lee JK. Local-pooled-error test for identifying differentially expressed genes with a small number of replicated microarrays. Bioinformatics. 2003 10 12;19(15):1945–51. 14555628 10.1093/bioinformatics/btg264

[13] Dudoit S, Yang YH, Callow MJ, Speed TP. Statistical methods for identifying differentially expressed genes in replicated cDNA microarray experiments. Statistica sinica. 2002 1 1;12(1):111–40.

[14] Klebanov L, Gordon A, Xiao Y, Land H, Yakovlev A. A permutation test motivated by microarray data analysis. Computational statistics & data analysis. 2006 8 31;50(12):3619–28.

[15] Benjamini Y, Hochberg Y. Controlling the false discovery rate: a practical and powerful approach to multiple testing. Journal of the Royal Statistical Society. Series B (Methodological). 1995 1 1:289–300.

[16] Kantoff PW, Schuetz TJ, Blumenstein BA, Glode LM, Bilhartz DL, Wyand M, et al Overall survival analysis of a phase II randomized controlled trial of a Poxviral-based PSA-targeted immunotherapy in metastatic castration-resistant prostate cancer. Journal of Clinical Oncology. 2010 3 1;28(7):1099–105. 10.1200/JCO.2009.25.0597 20100959 PMC2834462

[17] Newland RC, Dent OF, Lyttle MN, Chapuis PH, Bokey EL. Pathologic determinants of survival associated with colorectal cancer with lymph node metastases. A multivariate analysis of 579 patients. Cancer. 1994 4 15;73(8):2076–82. 8156513 10.1002/1097-0142(19940415)73:8<2076::aid-cncr2820730811>3.0.co;2-6

[18] Ogino S, Nosho K, Kirkner GJ, Kawasaki T, Meyerhardt JA, Loda M, et al CpG island methylator phenotype, microsatellite instability, BRAF mutation and clinical outcome in colon cancer. Gut. 2009 1 1;58(1):90–6. 10.1136/gut.2008.155473 18832519 PMC2679586

[19] Bauer KR, Brown M, Cress RD, Parise CA, Caggiano V. Descriptive analysis of estrogen receptor (ER)‐negative, progesterone receptor (PR)‐negative, and HER2‐negative invasive breast cancer, the so‐called triple‐negative phenotype. Cancer. 2007 5 1;109(9):1721–8. 17387718 10.1002/cncr.22618

[20] Wei LJ. The accelerated failure time model: a useful alternative to the Cox regression model in survival analysis. Statistics in medicine. 1992 1 1;11(14–15):1871–9. 1480879 10.1002/sim.4780111409

[21] Brennan AM, Mantzoros CS. Drug insight: the role of leptin in human physiology and pathophysiology—emerging clinical applications. Nature Reviews Endocrinology. 2006 6 1;2(6):318–27. 10.1038/ncpendmet0196 16932309

[22] Reiner-Benaim A, Yekutieli D, Letwin NE, Elmer GI, Lee NH, Kafkafi N, et al Associating quantitative behavioral traits with gene expression in the brain: searching for diamonds in the hay. Bioinformatics. 2007 9 1;23(17):2239–46. 17827208 10.1093/bioinformatics/btm300

[23] Park HR, Park M, Choi J, Park KY, Chung HY, Lee J. A high-fat diet impairs neurogenesis: involvement of lipid peroxidation and brain-derived neurotrophic factor. Neuroscience letters. 2010 10 4;482(3):235–9. 10.1016/j.neulet.2010.07.046 20670674

[24] Shapiro SS, Wilk MB. An analysis of variance test for normality (complete samples). Biometrika. 1965 12 1:591–611.

[25] Westfall PH, Young SS. Resampling-based multiple testing: Examples and methods for p-value adjustment. John Wiley & Sons; 1993 1 5.

[26] Zoico E, Zamboni M, Di Francesco V, Mazzali G, Fantin F, De Pergola G et al Relation between adiponectin and bone mineral density in elderly post-menopausal women: role of body composition, leptin, insulin resistance, and dehydroepiandrosterone sulfate. Journal of endocrinological investigation. 2008 4 1;31(4):297–302. 18475046 10.1007/BF03346361

[27] Iacobellis G, Cristina Ribaudo M, Zappaterreno A, Valeria Iannucci C, Leonetti F. Relationship of thyroid function with body mass index, leptin, insulin sensitivity and adiponectin in euthyroid obese women. Clinical endocrinology. 2005 4 1;62(4):487–91. 15807881 10.1111/j.1365-2265.2005.02247.x

[28] Osuna C JA, Gomez-Perez R, Arata-Bellabarba G, Villaroel V. Relationship between BMI, total testosterone, sex hormone-binding-globulin, leptin, insulin and insulin resistance in obese men. Systems Biology in Reproductive Medicine. 2006;52(5):355–61. 10.1080/01485010600692017 16873135

[29] Garfield AS, Cowley M, Smith FM, Moorwood K, Stewart-Cox JE, Gilroy K, et al Distinct physiological and behavioural functions for parental alleles of imprinted Grb10. Nature. 2011 1 27;469(7331):534–8. 10.1038/nature09651 21270893 PMC3031026

[30] Karanth S, Denovan‐Wright EM, Thisse C, Thisse B, Wright JM. The evolutionary relationship between the duplicated copies of the zebrafish fabp11 gene and the tetrapod FABP4, FABP5, FABP8 and FABP9 genes. FEBS journal. 2008 6 1;275(12):3031–40. 10.1111/j.1742-4658.2008.06455.x 18445037

[31] Esapa CT, Waite A, Locke M, Benson MA, Kraus M, McIlhinney RJ et al SGCE missense mutations that cause myoclonus-dystonia syndrome impair ε-sarcoglycan trafficking to the plasma membrane: modulation by ubiquitination and torsinA. Human molecular genetics. 2007 2 1;16(3):327–42. 17200151 10.1093/hmg/ddl472

[32] Mendell JR, Rodino‐Klapac LR, Rosales XQ, Coley BD, Galloway G, Lewis S, Malik V, Shilling C, Byrne BJ, Conlon T, Campbell KJ. Sustained alpha‐sarcoglycan gene expression after gene transfer in limb‐girdle muscular dystrophy, type 2D. Annals of neurology. 2010 11 1;68(5):629–38. 10.1002/ana.22251 21031578 PMC2970162

[33] Huang DW, Sherman BT, Lempicki RA. Systematic and integrative analysis of large gene lists using DAVID Bioinformatics Resources. Nature Protoc. 2009;4(1):44–57 19131956 10.1038/nprot.2008.211

[34] Huang DW, Sherman BT, Lempicki RA. Bioinformatics enrichment tools: paths toward the comprehensive functional analysis of large gene lists. Nucleic Acids Res. 2009;37(1):1–13 10.1093/nar/gkn923 19033363 PMC2615629

