# Correction: A Model-Based Joint Identification of Differentially Expressed Genes and Phenotype-Associated Genes

**DOI:** 10.1371/journal.pone.0153341

**Published:** 2016-04-05

**Authors:** The PLOS ONE Staff

There are errors in the Funding section. The publisher apologizes for these errors. The correct funding information is as follows: This work is supported by the National Research Foundation of Korea (NRF) grants funded by the Korean government (MSIP) GRANT numbers: 2015R1A5A001906 and 2013M3A9C4078158.

## References

[1] Cho SS, Kim Y, Yoon J, Seo M, Shin S-k, Kwon E-Y, et al (2016) A Model-Based Joint Identification of Differentially Expressed Genes and Phenotype-Associated Genes. PLoS ONE 11(3): e0149086 doi:10.1371/journal.pone.0149086 26964035 10.1371/journal.pone.0149086 PMC4786130

