# Supplementary material for: A Model-Based Joint Identification of Differentially Expressed Genes and Phenotype-Associated Genes
Source: PLoS One. 2016 Mar 10;11(3):e0149086. doi: 10.1371/journal.pone.0149086 (PMC4786130; doi:10.1371/journal.pone.0149086)
Supplement: S1 File — P-values for most genes are larger than 0.05 which means log-transformed gene expressions follow normal distributions (Fig A). QQ-plots for some genes which are significant in model-based approach (Fig B). Gene expression profiles for significant genes with leptin detected by model-based approach. Blue dots are ND samples and Red dots are HFD samples. X-axis shows log-normalized gene expression for each gene and Y-axis shows expression of leptin (Fig C). Gene expression profiles for significant genes with adiponectin detected by model-based approach. Blue dots show ND samples and Red dots show HFD samples. X-axis shows log-normalized gene expression for each gene and Y-axis shows expression of adiponectin (Fig D). Gene expression profiles for significant genes with insulin detected by model-based approach. Blue dots are ND samples and Red dots are HFD samples. X-axis shows log-normalized gene expression for each gene and Y-axis shows expression of insulin (Fig E). (PPTX) [file pone.0149086.s001.pptx]

## Slide 1
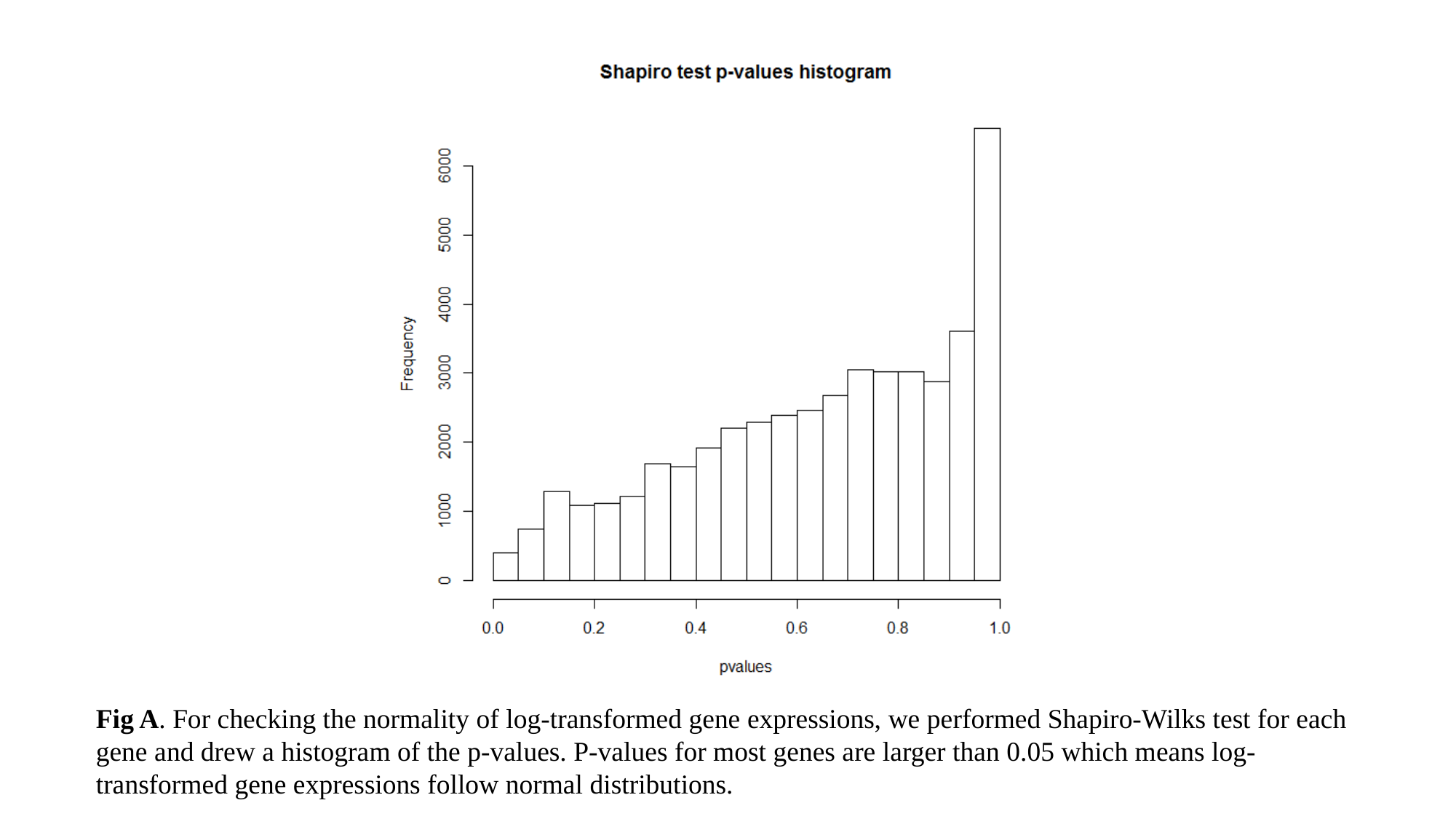

Fig A. For checking the normality of log-transformed gene expressions, we performed Shapiro-Wilks test for each gene and drew a histogram of the p-values. P-values for most genes are larger than 0.05 which means log-transformed gene expressions follow normal distributions.

## Slide 2
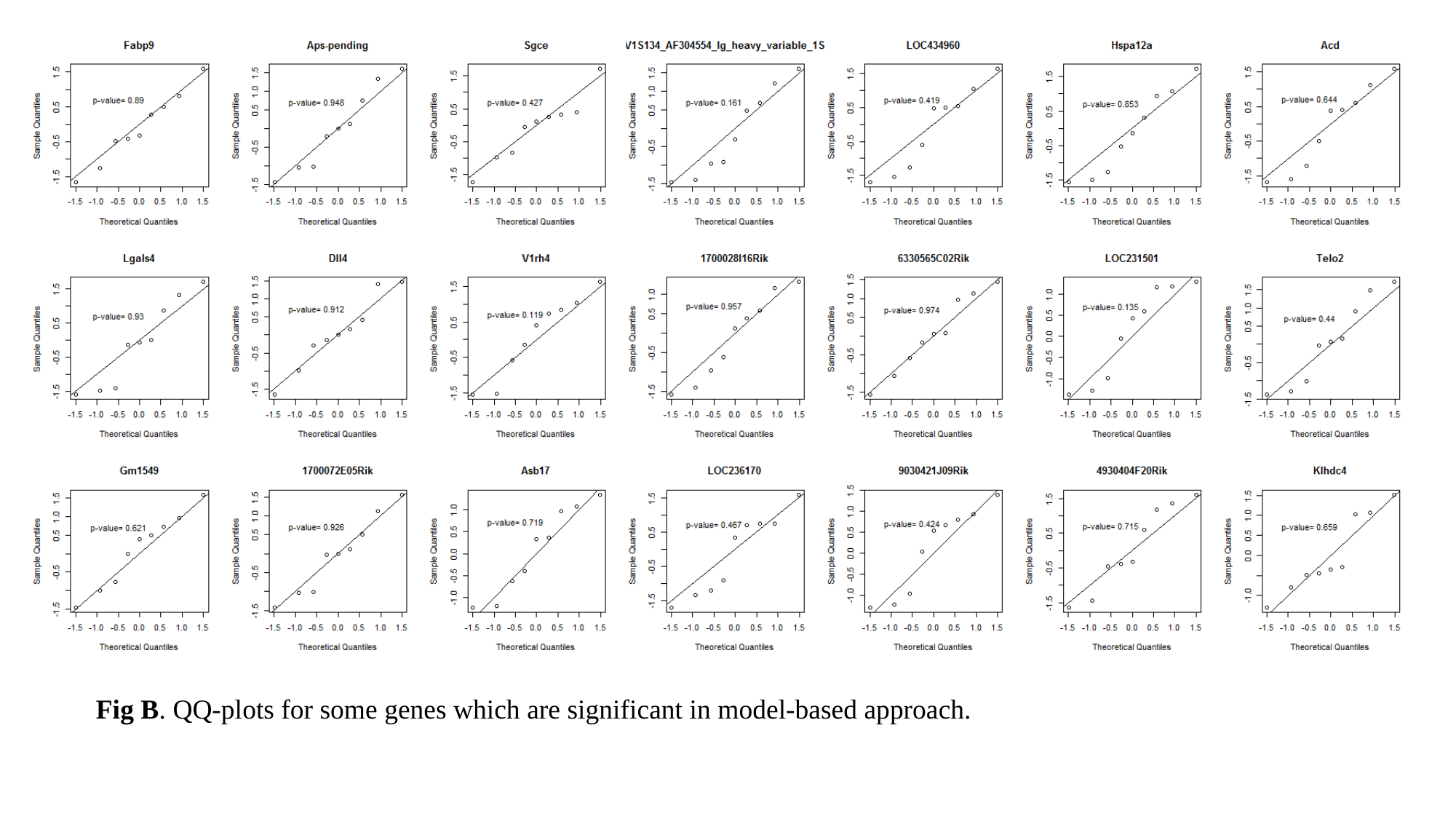

#
Fig B. QQ-plots for some genes which are significant in model-based approach.

## Slide 3
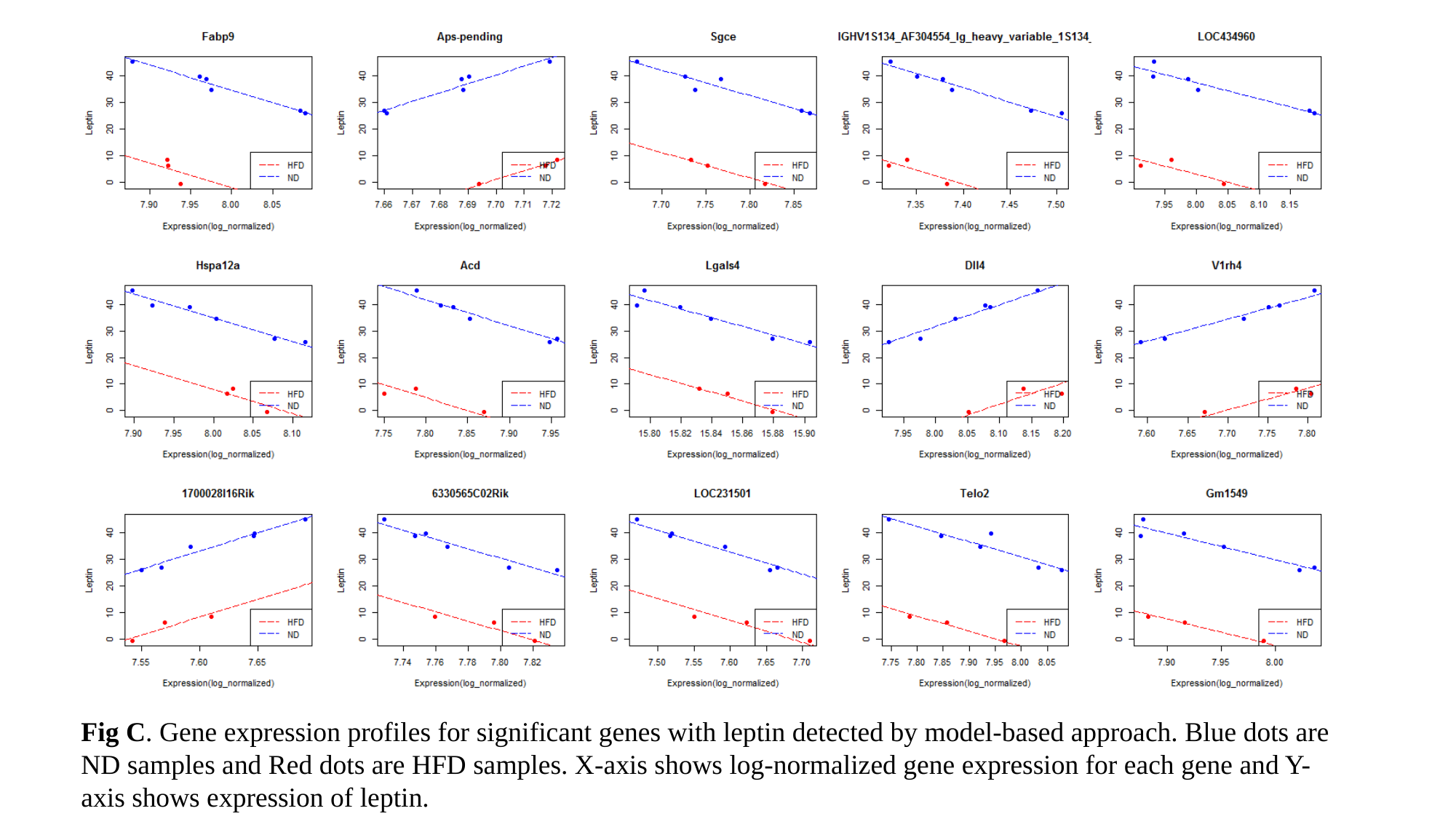

#
Fig C. Gene expression profiles for significant genes with leptin detected by model-based approach. Blue dots are ND samples and Red dots are HFD samples. X-axis shows log-normalized gene expression for each gene and Y-axis shows expression of leptin.

## Slide 4
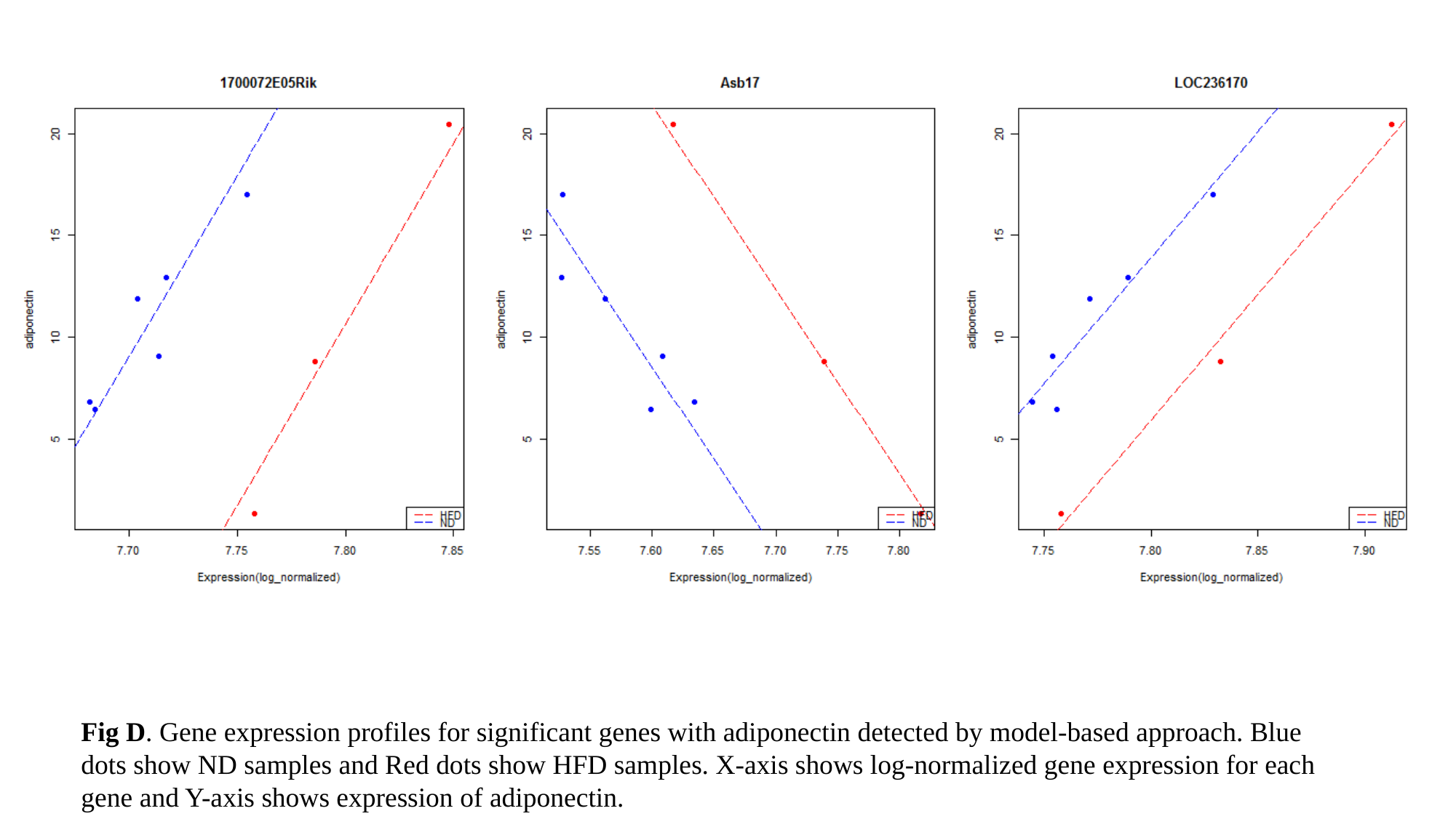

#
Fig D. Gene expression profiles for significant genes with adiponectin detected by model-based approach. Blue dots show ND samples and Red dots show HFD samples. X-axis shows log-normalized gene expression for each gene and Y-axis shows expression of adiponectin.

## Slide 5
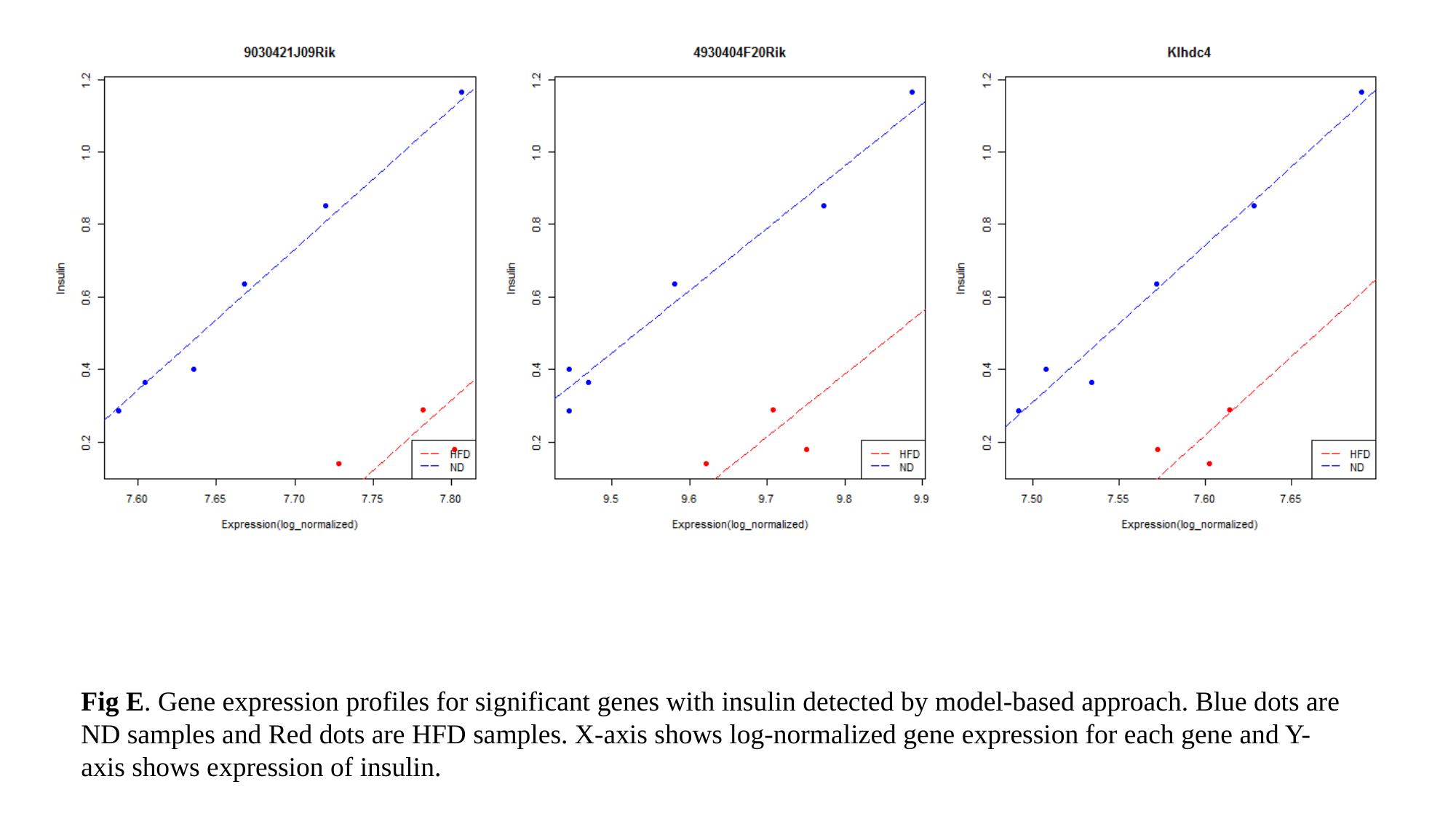

#
Fig E. Gene expression profiles for significant genes with insulin detected by model-based approach. Blue dots are ND samples and Red dots are HFD samples. X-axis shows log-normalized gene expression for each gene and Y-axis shows expression of insulin.
